# Computing the Dissociation Constant from Molecular Dynamics Simulations with Corrections for the Large Pressure Fluctuations—Aquaglyceroporins Have High Affinity for Their Substrate Glycerol

**DOI:** 10.3390/biom16010174

**Published:** 2026-01-21

**Authors:** Md Mohsin, Hans R. Loja, Liao Y. Chen

**Affiliations:** 1 Department of Physics, The University of Texas at San Antonio, One UTSA Circle, San Antonio, TX 78249, USA; md.mohsin@my.utsa.edu; 2 Department of Integrative Biology, The University of Texas at San Antonio, San Antonio, TX 78249, USA; hans.loja@my.utsa.edu

**Keywords:** affinity, aquaglyceroporin, molecular dynamics

## Abstract

In this paper, we consider the inevitable large fluctuations of pressure in typical molecular dynamics (MD) simulations of ligand–protein binding problems. In simulations under the constant pressure of one bar, the pressure artifactually fluctuates over the range of ±100 bars or more. This artifact can cause gross inaccuracy in the apparent binding affinity computed as the ratio of the probability for the ligand to be bound inside the protein and the probability for the ligand to be outside the protein. Based on statistical thermodynamics, we derive a correction factor for the ligand–protein binding affinity to compensate for the artifactual pressure fluctuations. The correction factor depends on the change in the system volume between the bound and the unbound states of the ligand. We conducted four sets of MD simulations for glycerol affinities with four aquaglyceroporins: AQP10, AQP3, AQP7, and GlpF. Without the correction factor, the apparent affinity of glycerol with each of these four aquaglyceroporins is computed directly from the simulations to be very low (~1/M). With the correction factor applied, glycerol’s affinity is computed to be 1/mM to 1/µM. In conclusion, glycerol has high affinity for its native facilitator aquaglyceroporins, which is in contrast to the current literature not correcting the artifactual consequences of the large pressure fluctuations in typical in silico experiments.

## 1. Introduction

**Figure 1:**
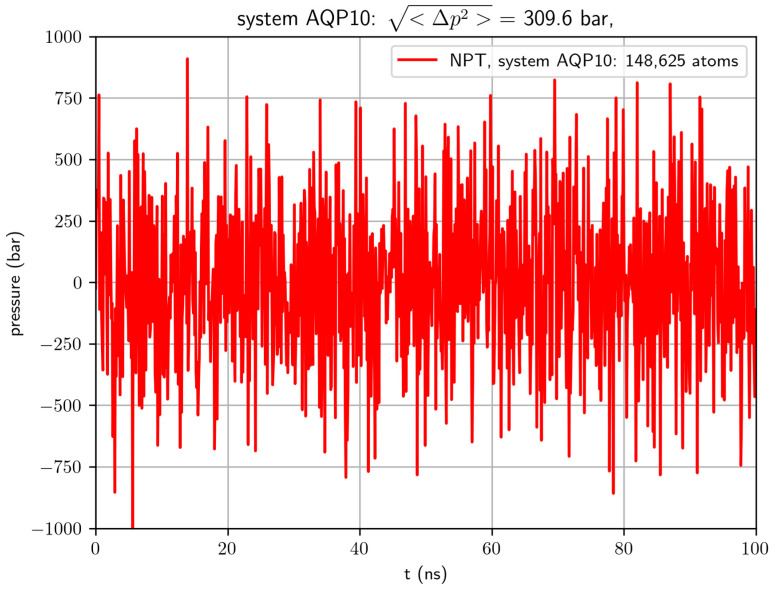
Pressure fluctuation in an all-atom NPT (constant number of atoms *N* = 148,625, constant pressure *P* = 1 bar, and constant temperature *T* = 303 K) simulation of an AQP10 tetramer embedded in a lipid bilayer situated in the center of a physiological saline box.

The determination of the binding affinity (or its inverse, the dissociation constant) of a protein for its substrate molecule is fundamental to the understanding of biophysical/biochemical processes. In principle, binding affinity can simply be computed from molecular dynamics (MD) simulations by counting the protein–substrate binding and unbinding events from the simulated dynamics. However, typical simulations of a system consisting of a few hundred thousand atoms are not suitable for such a simple task, for the following reason: A typical model system carries fluctuations in pressure around a few hundred bars for an NPT simulation (N indicates constant number of molecules in a model system, *P* means a constant pressure of one bar, and *T* represents a constant temperature). See Figure 1 for an example. Based on statistical thermodynamics [1], this undesirable artifact is inevitable due to the smallness of the system size (as explicitly pointed out in NAMD User’s Guide https://www.ks.uiuc.edu/Research/namd/2.14/ug/node39.html (accessed on 28 December 2025)), the pressure fluctuations are inversely proportional to the volume of a model system). This huge pressure fluctuation naturally favors unbinding protein from its substrate and thus leads to the computed affinity (apparent affinity) being significantly lower than the true affinity [2]. In other words, the computed apparent dissociation $k_{D}^{app}$ can be significantly larger than the true dissociation constant $k_{D}$.

In this paper, we take a theoretical consideration of the artifactual pressure fluctuations and derive a correction factor for the computed apparent dissociation constant to estimate the true $k_{D}$. We run four sets of MD simulations for the binding affinities of four aquaglyceroporins (AQGPs) (human AQP10, AQP3, AQP7, and *E. coli* GlpF) for their substrate glycerol. In each case, the apparent affinity computed from the simulation is very low ($k_{D}^{app}$~100 mM) but the corrected estimation for the true $k_{D}$ is in the µM range. This confirms that aquaglyceroporins have reasonably high affinities for their substrate glycerol.

The significance of this study stems from the following fact: AQGPs, constituting a subfamily of aquaporin (AQP) proteins [3,4,5], play essential roles in human physiological processes. They facilitate the transport of glycerol and some other nutrient molecules across the cell membrane [6], which are fundamental to many physiological/pathophysiological processes such as fat metabolism and metabolic diseases [7]. This research produces insights into two controversies about whether or not an aquaglyceroporin has high affinity for its substrate glycerol and why typical simulations suggest very low aquaglyceroporin–glycerol affinity. Here is a brief summary of the current state: In 1994, in vitro experiments showed that *E. coli* aquaglyceroporin GlpF facilitates the unsaturable uptake of glycerol up to 200 mM into Xenopus oocytes [8]. These experiments suggested that GlpF has very low affinity for its substrate glycerol. From 2008 to 2014, however, multiple experiments [9,10,11] demonstrated that human aquaglyceroporins AQP7, AQP9, and AQP10 conduct the saturated transport of glycerol. In these experiments, the Michaelis constants were measured to be around 10 µM, indicating that human AQGPs have high affinities for glycerol. Examining the crystal structures of AQGPs (GlpF in 2000 [12], *P. falciparum* PfAQP in 2008 [13], AQP10 in 2018 [14], and AQP7 in 2020 [15,16,17]), they all have glycerol molecules inside the AQGP channel and near the channel openings on both the intracellular (IC) and the extracellular (EC) sides. These structures tell us that all four AQGPs have affinities for glycerol. If we insisted that unsaturated transport precludes high affinity, the previously stated data would suggest inconsistency among the experiments in the current literature. However, in an in silico in vitro study [18] of glycerol uptake into human erythrocytes through AQP3 [19], we showed that “an AQGP (having high affinity for its substrate glycerol) can conduct glycerol transport that is unsaturated up to 400 mM. The transport pathway for unsaturated transport through a high affinity facilitator protein was shown to involve two glycerol molecules next to each other, both bound inside an AQP3 channel (one at the high affinity site and one at a low affinity site) for the transport of one glycerol molecule across the cell membrane [18]. It is the substrate–substrate interactions (mostly repulsion due to steric exclusion) inside a single-file channel that make it easy for two glycerol molecules cooperatively to move one substrate molecule across the AQGP channel via the high affinity site.” Therefore, in vitro experiments exhibiting unsaturated glycerol transport do not preclude high affinity between an AQGP and its substrate glycerol. All in vitro experiments either allow or show the AQGP–glycerol affinity being high. This study demonstrates why the simulations of the current literature fail to predict such high affinities and how to correct for the artifactual large fluctuations in typical MD studies.

## 2. Methods

### 2.1. Model System Setup and Simulation Parameters

We followed the well-tested steps in the literature to set up our model systems and to run MD simulations. Therefore, this subsection is mostly direct quotes from the current literature with minor adaptations: “We employed CHARMM-GUI [20,21,22] to build an all-atom model of an AQGP tetramer embedded in a 120 Å × 120 Å patch of membrane (lipid bilayer consisting of 193 phosphatidylethanolamine/POPE, 119 phosphatidylcholine/POPC, and 80 cholesterol/CHL1 molecules). The coordinates of AQP10 (PDB: 6F7H), AQP3, AQP7 (PDB: 6QZI), and GlpF (PDB: 1FX8) were taken from Ref. [14], Ref. [23], Ref. [15] and Ref. [12], respectively. The positioning of the AQGP tetramer was determined by matching the hydrophobic side surface with the lipid tails and aligning the channel axes perpendicular to the membrane. The AQGP–membrane complex was sandwiched between two layers of TIP3P waters, each of which was approximately 30 Å thick. The system was then neutralized and salinated with Na^+^ and Cl^−^ ions to a salt concentration of 150 mM. Finally, glycerol was added to the system to a concentration of $c_{G}$. In this way, four all-atom model systems were built for the four AQGPs: System AQP10, System AQP3, System AQP7, and System GlpF.

We employed NAMD 2.13 (for initial stage of a simulation when constraints were needed) and NAMD 3.0 (for all equilibrium runs when no constraints were used) [24,25] as the MD engines. We used CHARMM36 parameters [26,27,28] for inter- and intra-molecular interactions. We followed the literature’s standard steps to equilibrate the system [17,29,30,31]. Then, we ran unbiased MD for a few thousand ns with constant pressure at 1.0 bar (Nose–Hoover barostat) and constant temperature at 303.15 K (Langevin thermostat). The Langevin damping coefficient was chosen to be 1/ps. The Langevin piston period was 50 fs and the Langevin piston decay was 25 fs. The periodic boundary conditions were applied to all three dimensions. The particle mesh Ewald (PME) was used for the long-range electrostatic interactions (grid level: 128 × 128 × 128). The time step was 2.0 fs. The cut-off for long-range interactions was set to 10 Å with a switching distance of 9 Å.”

### 2.2. Direct Computation of Apparent Affinity from MD Simulations

**Figure 2:**
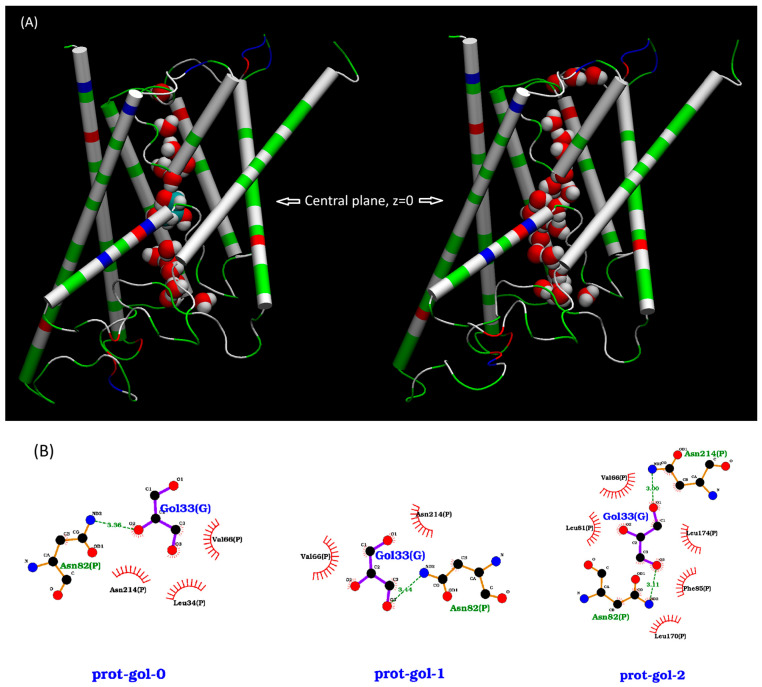
(**A**) Water molecules inside an AQP10 channel with (**left**) and without (**right**) a glycerol molecule residing inside the channel near the NAA/NPS motifs. The AQP10 protein is shown in cartoons colored by residue types (hydrophilic, green; hydrophobic, white; positively charged, blue; negatively charged, red); water and glycerol molecules in spheres colored by atoms (oxygen, red; hydrogen, white; carbon, cyan). The top side is the IC side and the bottom, the EC side. The NAA/NPS motifs are where the two half transmembrane helices meet. (**B**) 2D illustration of glycerol–protein interactions from three representative frames of an MD trajectory when glycerol is near the NAA/NPS motifs. In this paper, we employed VMD [32] to generate 3D molecular graphics and LigPlot+ [33] to render 2D illustrations, respectively.

We ran the MD simulation for each system for a sufficiently long time. After a system was fully equilibrated, we continued the MD run for hundreds of ns and used that part of the MD trajectory to compute the probability $p_{b}$ for an AQGP channel being occupied with a glycerol molecule. We considered a channel as being occupied when a glycerol molecule was found in the single-file region of the channel, 7.1 Å to the IC/EC side from the NAA/NPS motifs. We illustrate such a case of occupancy in the left panel of Figure 2. When the system is in its equilibrium state, the probability $p_{b}=c_{G}/(c_{G}+k_{D})$ for a given glycerol concentration $c_{G}$, from which we computed the dissociation constant from the binding probability: $k_{D}^{app.}=c_{G}(1-p_{b})/p_{b}$.

### 2.3. Theoretical Formulation of Correction for the Pressure Fluctuation

The dissociation constant of a substrate–protein complex can be computed from two partial partitions:

$$k_{D}=c_{0}\frac{Z_{\infty }(N,P,T)}{Z_{0}(N,P,T)}.$$

Here, *N* is the number of molecules in the system; *P*, the pressure; and *T*, the absolute temperature. The partial partition $Z_{\infty }$ is the NPT ensemble partition of the system under the condition that the substrate molecule is away from the protein, freely sampling the volume of $\frac{1}{c_{0}}$ where $c_{0}$ is the standard concentration of 1 mol/L. The partial partition $Z_{0}$ is the NPT ensemble partition of the system under the condition that the substrate and the protein are bound together as a complex. In this, the two partial partitions have identical dimensionalities and units. In an in silico implementation of the NPT ensemble, the preservation of *N* constant is trivial and the maintenance of T constant is well accomplished with fluctuations much smaller than the temperature *T* but the fluctuations in *P* are much greater than the constant pressure of $p_{0}=1$ bar in a typical MD study of the literature (See, e.g., Figure 1). The root mean square of the pressure fluctuations $\Delta P=\sqrt{<{\left(P-p_{0}\right)}^{2}>}$ is much greater than the mean pressure $<P>=p_{0}$. (The brackets represent statistical average over *P*.) Therefore, the computed value, the apparent dissociation constant,

$$k_{D}^{app.}=c_{0}\frac{{<Z}_{\infty }\left(N,P,T\right)>}{{<Z}_{0}\left(N,P,T\right)>}\;$$

which is not equal to the true dissociation constant

$$k_{D}=c_{0}\frac{Z_{\infty }(N,P=p_{0},T)}{Z_{0}(N,P=p_{0},T)}.$$

Consider approximating the Gibbs free energy $G\left(N,P,T\right)=-k_{B}T\ln Z(N,P,T)$ as follows ($k_{B}$ denoting the Boltzmann constant):

$$G\left(N,P,T\right)=G\left(N,p_{0},T\right)+\left(P-p_{0}\right)V.$$

Here, V=∂G/∂P is the mean volume of the system. Assuming the simplest form of the pressure fluctuations (with a Gaussian distribution), we have the following approximations for the partial partitions:

$${<Z}_{\infty }\left(N,P,T\right)>=<e^{-G_{\infty }(N,P,T)/k_{B}T}>=Z_{\infty }(N,p_{0},T)<e^{-\left(P-p_{0}\right)V_{\infty }/k_{B}T}>,$$

$${<Z}_{0}\left(N,P,T\right)>=<e^{-G_{0}(N,P,T)/k_{B}T}>=Z_{0}\left(N,p_{0},T\right)<e^{-\left(P-p_{0}\right)V_{0}/k_{B}T}>.$$

Therefore, we arrive at the following relationship:

$$k_{D}^{app.}=k_{D}<e^{-\left(P-p_{0}\right)V_{\infty }/k_{B}T}>/<e^{-\left(P-p_{0}\right)V_{0}/k_{B}T}>.$$

Solving for $k_{D}$ and carrying out the Gaussian averages, we obtain the following:

$$k_{D}{/\;k}_{D}^{app.}=\frac{e^{\frac{1}{2}<\Delta P^{2}>{\left(\frac{V_{0}}{k_{B}T}\right)}^{2}}}{e^{\frac{1}{2}<\Delta P^{2}>{\left(\frac{V_{\infty }}{k_{B}T}\right)}^{2}}}=e^{\frac{<\Delta P^{2}>}{2k_{B}^{2}T^{2}}(V_{0}^{2}-V_{\infty }^{2})}.$$

Here, $V_{0}$ and $V_{\infty }$ are, respectively, the volume of system when the substrate and the protein are bound together and the volume of the system when the substrate is away from the protein. Further, we can rewrite the above equation as

$$k_{D}{/\;k}_{D}^{app.}=e^{-\frac{<\Delta P^{2}>V}{k_{B}^{2}T^{2}}(V_{\infty }-V_{0})}.$$

It is a fundamental law of statistical thermodynamics [34] that $<\Delta P^{2}>≃k_{B}T/\beta V$ where β=−∂V/V∂P is the bulk modulus of the system. Then, we observe that the multiplier

$$k_{D}{/\;k}_{D}^{app.}≃e^{-(V_{\infty }-V_{0})/\beta k_{B}T}$$

can be easily evaluated from in silico simulations. This multiplier is the central result of this research, the correction factor for an accurate estimation of the true dissociation constant from simulations with large pressure fluctuations.

Considering $\beta \sim 5\times 10^{-10}/Pa$, $k_{B}T\sim 0.6\;kcal/mol$ and the small volume difference, e.g., $V_{\infty }-V_{0}=10\;mL/mol$, leads to $k_{D}{/\;k}_{D}^{app.}≃3.5\times 10^{-4}$. Indeed, the apparent dissociation constant computed directly from MD simulations is generally very far from the true dissociation constant, depending on the volume change when a substrate molecule is dissociated from the protein.

## 3. Results

**Figure 3:**
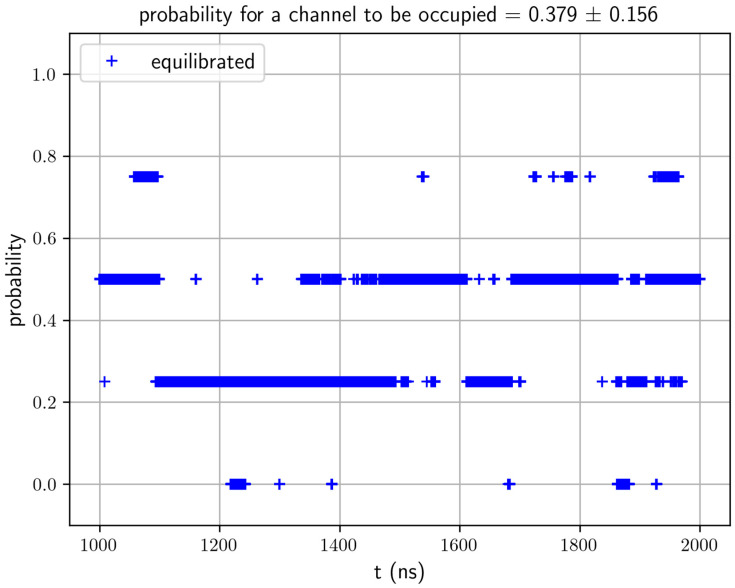
Probability for an AQP10 channel to be occupied by glycerol during the last 1000 ns of a 2000 ns MD run.

**Figure 4:**
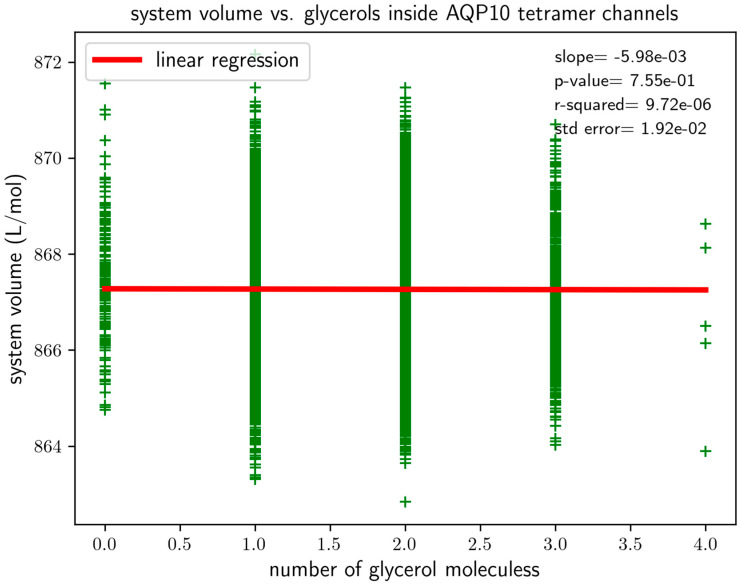
The volume of the system in correlation with the number of glycerol molecules being inside the protein.

We ran 2000 ns of MD simulation of System AQP10 (illustrated in SI, Figure S1) after the initial runs for system preparation/setup. The root mean square deviation (RMSD) curves of AQP10 tetramers (shown in SI, Figure S2) show that System AQP10 reached equilibrium during last 1000 ns of the simulation. All atoms of a protomer were included in the RMSD calculations. Therefore, we conducted equilibrium statistical analyses of data from this part of the MD for the estimation of glycerol–AQP10 affinity. In Figure 3, we plotted the probability for an AQP10 channel to be occupied by one or more glycerol molecules. This probability gives the apparent dissociation constant $k_{D}^{app.}=98.2\;mM$, suggesting low affinity for glycerol–AQP10, in line with the current literature. Also, for the last 1000 ns of the simulation, we analyzed the correlation between the volume of System AQP10 and number of glycerol molecules located inside the four channels of AQP10, which is shown in Figure 4. This analysis is important because a glycerol molecule displaces different numbers of water molecules when it is inside an AQP10 channel and when it is outside the protein (in the bulk of the saline). Consequently, the volume of System AQP10 fluctuates around different mean values. Linear regression of the data gives rise to the volume difference $V_{\infty }-V_{0}=5.98\;mL/mol$ (which is equal to the negative of the linear regression slope). Namely, the system volume averages higher by 5.98 mL/mol when a glycerol molecule is relocated from the bulk outside AQP10 to inside an AQP10 channel and, correspondingly, a number of water molecules move out of the channel to the bulk outside AQP10. The positive volume difference reflects on the fact that glycerol displaces fewer water molecules inside a channel than when it is outside the channel, which is illustrated in Figure 2. Using this volume difference in the correction factor, we obtain the estimated true dissociation constant $k_{D}=0.85\;mM$, demonstrating that AQP10 has reasonably high affinity for its substrate glycerol.

In studies similar to System AQP10, we conducted a 2600 ns MD run for System AQP3, a 1500 ns MD run for System AQP7, and a 1000 ns run for System GlpF. The simulation data are shown in SI, Figures S3–S14. In each case, the MD was run for at least 500 ns after the system reached full equilibrium, as indicated by the RMSD curves for the four monomers of an AQGP tetramer (SI, Figure S4 for System AQP3, Figure S8 for System AQP7, and Figure S12 for System GlpF). The last equilibrium parts of the trajectories (the last 600 ns for System AQP3, the last 500 ns for System AQP7, and the last 500 ns for System GlpF) were used to extract the probability for a glycerol molecule to be inside an AQGP channel (shown in SI, Figure S5 for System AQP3, Figure S9 for System AQP7, and Figure S13 for System GlpF), from which the apparent dissociation constants were computed. The results are tabulated in Table 1. The values of the apparent dissociation constant $\left(k_{D}^{app.}\right)$ all suggest very weak affinity between an AQGP and its substrate glycerol, which, again, is in line with the current literature. These equilibrium parts were further analyzed for the correlations between the system volume and the number of glycerol molecules inside an AQGP tetramer, which are shown in SI, Figure S6 for System AQP3, Figure S10 for System AQP7, and Figure S14 for System GlpF. Linear regression produces a negative slope, giving rise to a positive volume difference $V_{\infty }-V_{0}>0$ in each of the three cases (tabulated in Table 1) and producing an estimated true $k_{D}$ in micro molars.

## 4. Conclusions

Pressure fluctuations in a typical in silico study of the current literature can lead to predictions of very low affinities for protein–substrate binding problems. The apparent dissociation constant can be many times greater than the true dissociation constant if dissociation of the substrate from the protein corresponds to an increase in the system’s volume. The in silico studies of four aquaglyceroporins (human AQP10, AQP3, AQP7, and *E. coli* GlpF) all suggest low affinities without the correction factor to count for the large artifactual fluctuations in pressure. Once the correction factor is included, the estimated values of the true dissociation constant all lead to the conclusion that an aquaglyceroporin has reasonably high to very high affinity for its substrate glycerol.

## Figures and Tables

**Table 1:** Glycerol affinity estimated from MD simulations.

System (Glycerol Concentration)	$\mathrm{Apparent}\;k_{D}^{app.}$	$V_{\infty }-V_{0}$	$\mathrm{True}\;k_{D}$ Estimated
AQP10 (PDB: 6F7H) (60.0 mM)	98.2 mM	5.98 mL/mol	0.85 mM
AQP3 (computed) (435.3 mM)	431.8 mM	12.0 mL/mol	31.56 µM
AQP7 (PDB: 6QZI) (83.1 mM)	25.11 mM	14.4 mL/mol	0.27 µM
GlpF (PDB: 1FX8) (49.4 mM)	49.38 mM	10.2 mL/mol	15.05 µM

## Data Availability

The original contributions presented in this study are included in the article/Supplementary Materials. Further inquiries can be directed to the corresponding authors.

## Supplementary Materials

The following supporting information can be downloaded at https://www.mdpi.com/article/10.3390/biom16010174/s1, Figure S1 illustrates the all-atom model of AQP10 in a lipid bilayer fully solvated in a physiological saline; Figure S2 shows the RMSD of the AQP10 tetramers during the 2000 ns run of molecular dynamics, demonstra ng equilibrium behavior during the last 1000 ns; Figure S3 shows the pressure fluctua ons of System AQP3; Figure S4 shows the RMSD of the AQP3 tetramers during the 2600 ns run of molecular dynamics, demonstra ng equilibrium behavior during the last 1000 ns; Figure S5 shows the glycerol occupancy inside AQP3; Figure S6 shows the correla on between the system volume and the glycerol occupancy in System AQP3; Figure S7 shows the pressure fluctua ons of System AQP7; Figure S8 shows the RMSD of the AQP7 tetramers during the 1500 ns run of molecular dynamics, demonstra ng equilibrium behavior during the last 500 ns; Figure S9 shows the glycerol occupancy inside AQP7; Figure S10 shows the correla on between the system volume and the glycerol occupancy in System AQP7; Figure S11 shows the pressure fluctua ons of System GlpF; Figure S12 shows the RMSD of the GlpF tetramers during the 1000 ns run of molecular dynamics, demonstrating equilibrium behavior during the last 500 ns; Figure S13 shows the glycerol occupancy inside GlpF; Figure S14 shows the correlation between the system volume and the glycerol occupancy in System GlpF.
