# Supplementary material for: Computing the Dissociation Constant from Molecular Dynamics Simulations with Corrections for the Large Pressure Fluctuations—Aquaglyceroporins Have High Affinity for Their Substrate Glycerol
Source: Biomolecules. 2026 Jan 21;16(1):174. doi: 10.3390/biom16010174 (PMC12839012; doi:10.3390/biom16010174)
Supplement: Supplementary file 1 [file biomolecules-16-00174-s001.zip › biomolecules-3954174-supplementary.pdf]

Supplemental Information to

Computing the dissociation constant from molecular dynamics simulations with corrections for the large pressure fluctuations---aquaglyceroporins have high affinity for their substrate glycerol

Md Mohsin[1], Hans R Loja[2] and Liao Y Chen[1]*

In this supplemental information, we provide the following details that are needed to reproduce the research presented in the main text:

Fig. S1 illustrates the all-atom model of AQP10 in a lipid bilayer fully solvated in a physiological saline.

Fig. S2 shows the RMSD of the AQP10 tetramers during the 2,000 ns run of molecular dynamics, demonstrating equilibrium behavior during the last 1,000 ns.

Fig. S3 shows the pressure fluctuations of System AQP3.

Fig. S4 shows the RMSD of the AQP3 tetramers during the 2,600 ns run of molecular dynamics, demonstrating equilibrium behavior during the last 1,000 ns.

Fig. S5 shows the glycerol occupancy inside AQP3.

Fig. S6 shows the correlation between the system volume and the glycerol occupancy in System AQP3.

Fig. S7 shows the pressure fluctuations of System AQP7.

Fig. S8 shows the RMSD of the AQP7 tetramers during the 1,500 ns run of molecular dynamics, demonstrating equilibrium behavior during the last 500 ns.

Fig. S9 shows the glycerol occupancy inside AQP7.

Fig. S10 shows the correlation between the system volume and the glycerol occupancy in System AQP7.

Fig. S11 shows the pressure fluctuations of System GlpF.

Fig. S12 shows the RMSD of the GlpF tetramers during the 1,000 ns run of molecular dynamics, demonstrating equilibrium behavior during the last 500 ns.

Fig. S13 shows the glycerol occupancy inside GlpF.

Fig. S14 shows the correlation between the system volume and the glycerol occupancy in System GlpF.

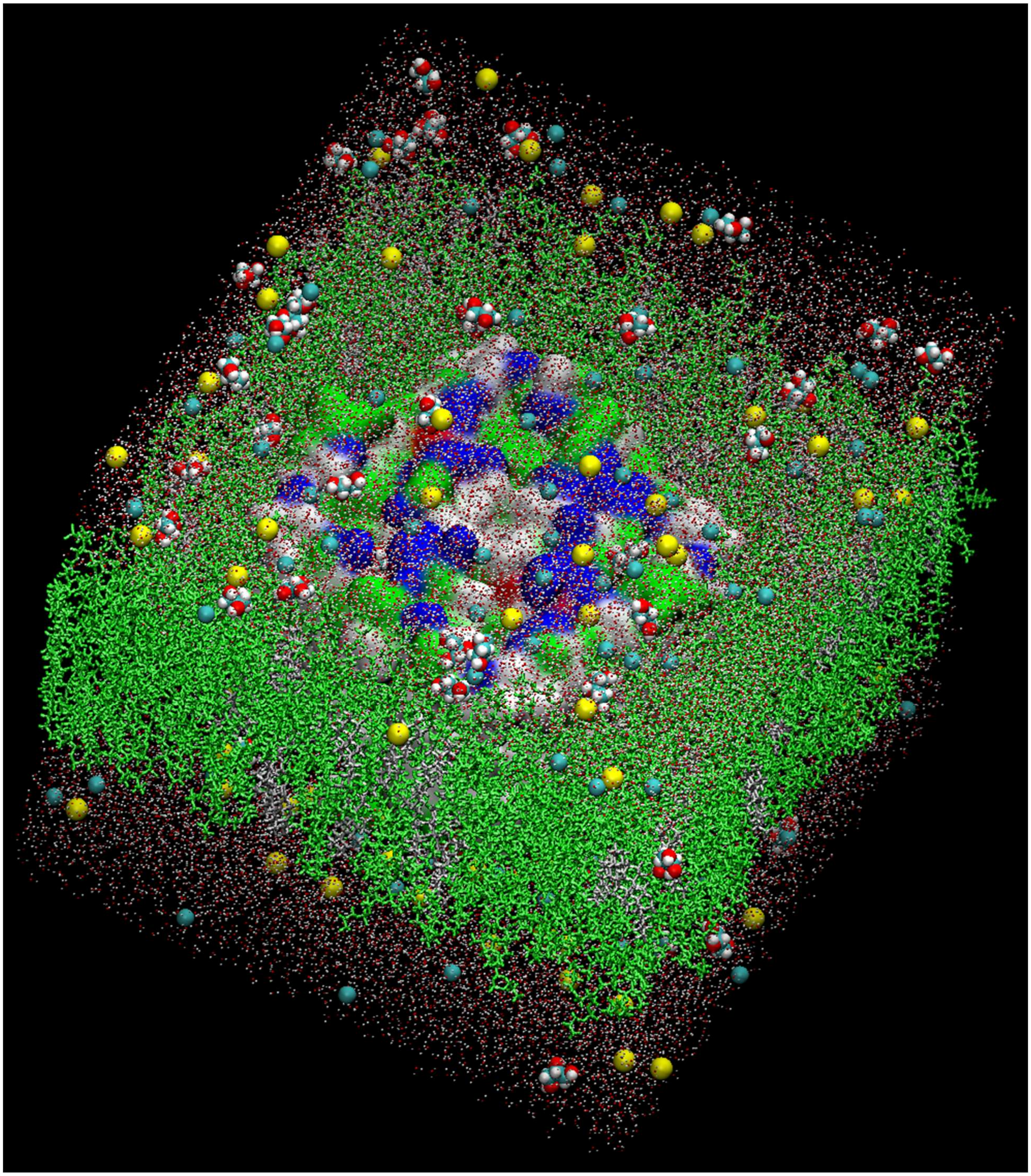

Fig. S1. All-atom model: System AQP10 consists of AQP10 tetramer (shown in surface colored by residue types: hydrophillic, green; hydrophobic, white; positively charged, blue; negatively charged, red) whose His residues are neutral at pH7, 29,877 TIP3P water molecules (red-and-white dots), 312 POPE/POPC lipids (green licorices), 80 cholestrol molecules (gray licorices), 86 chloride anions (cyan balls), 82 sodium ions (yellow balls), and 52 glycerol molecules (red-and-white-and-cyan balls).

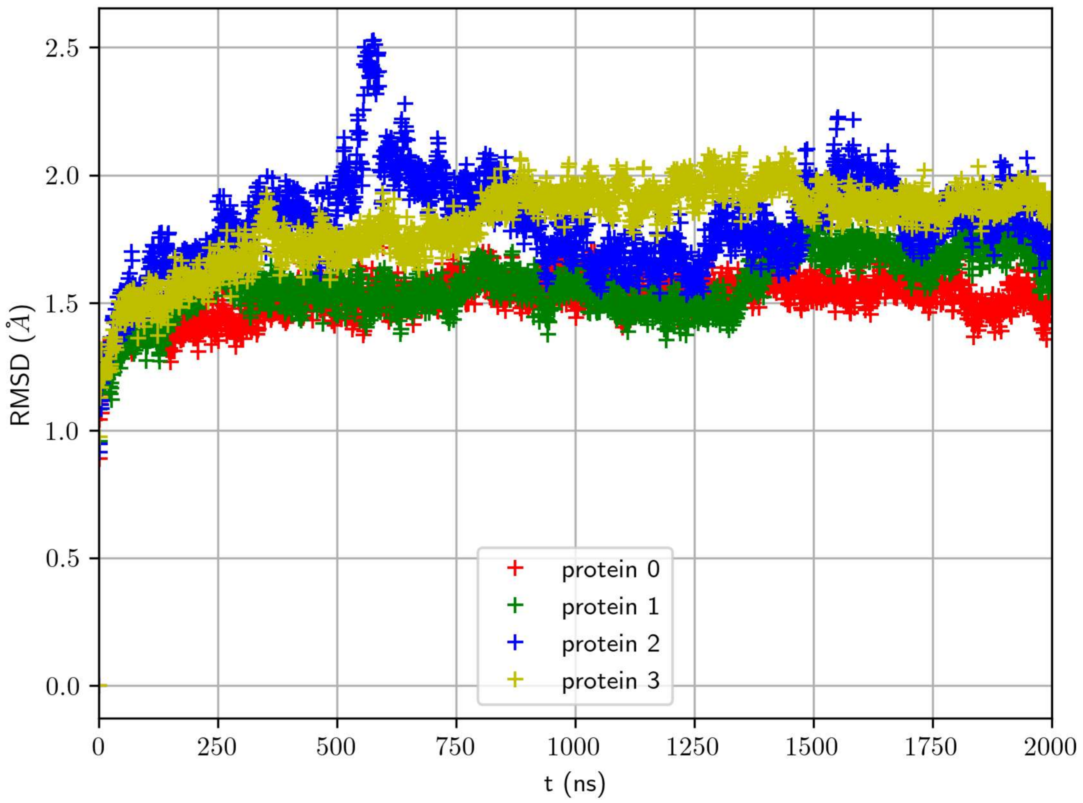

Fig. S2. RMSD of each of the tetramer proteins in System AQP10.

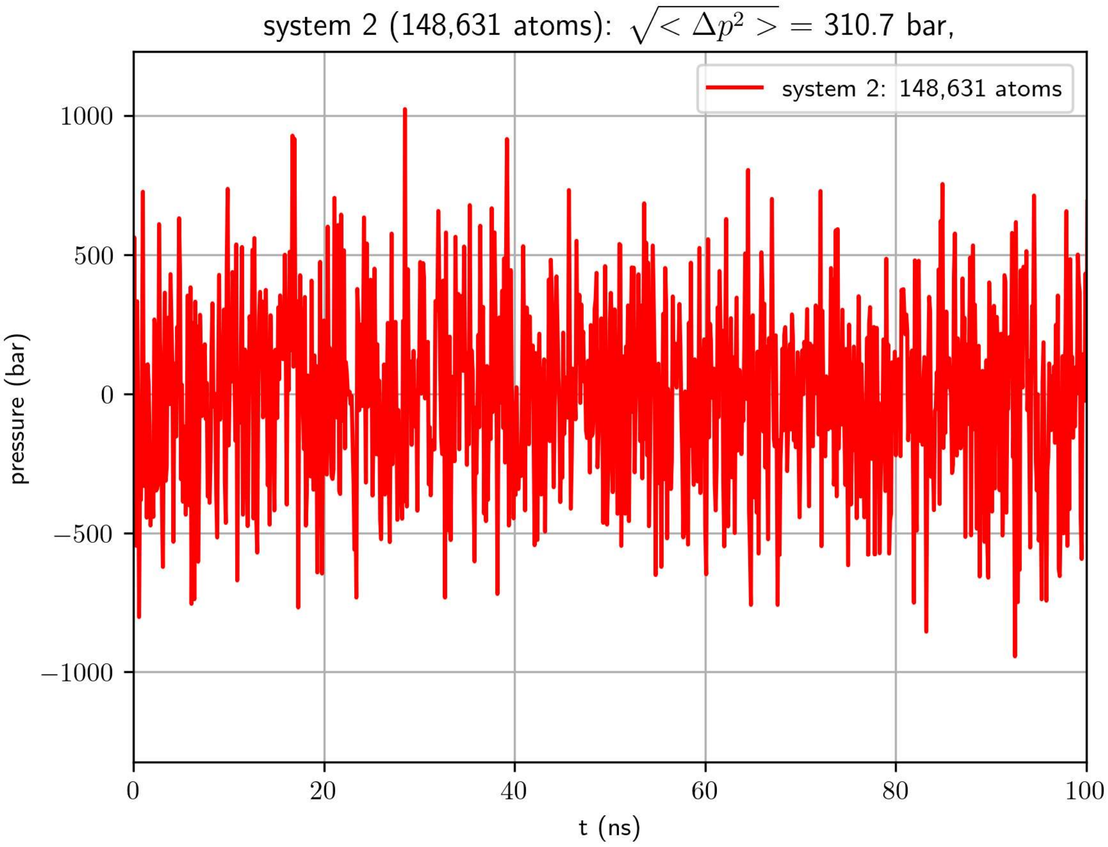

Fig. S3. Pressure fluctuation of System AQP3.

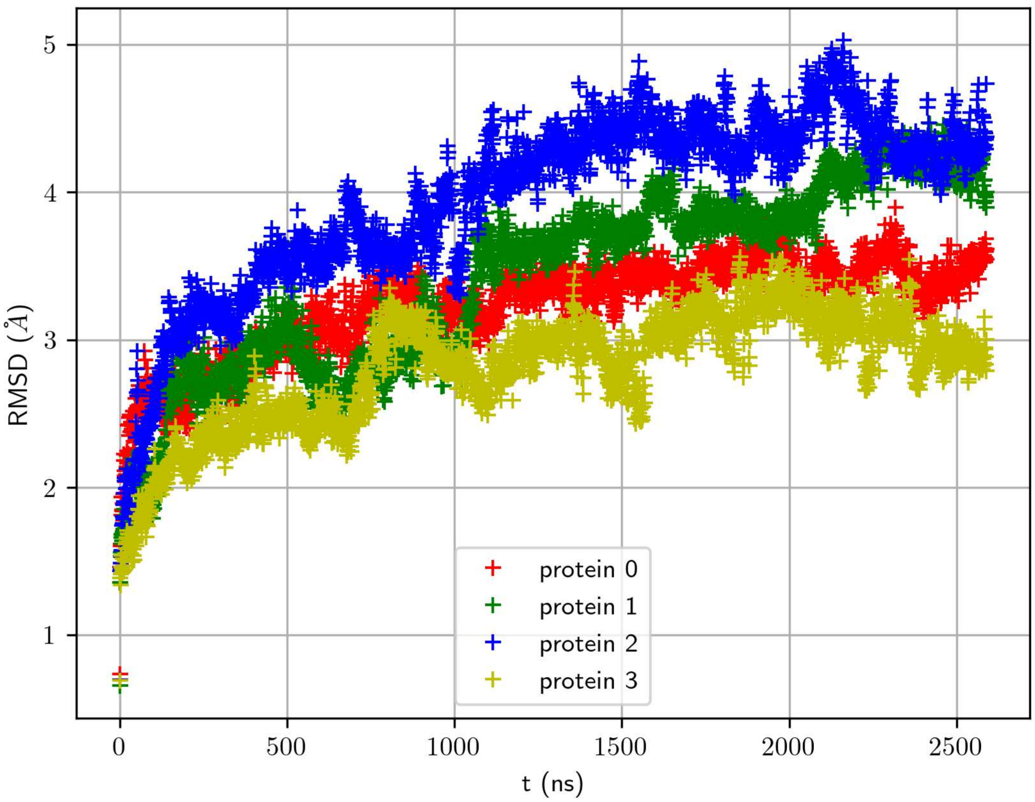

Fig. S4. RMSD of each of the tetramer proteins in System AQP3.

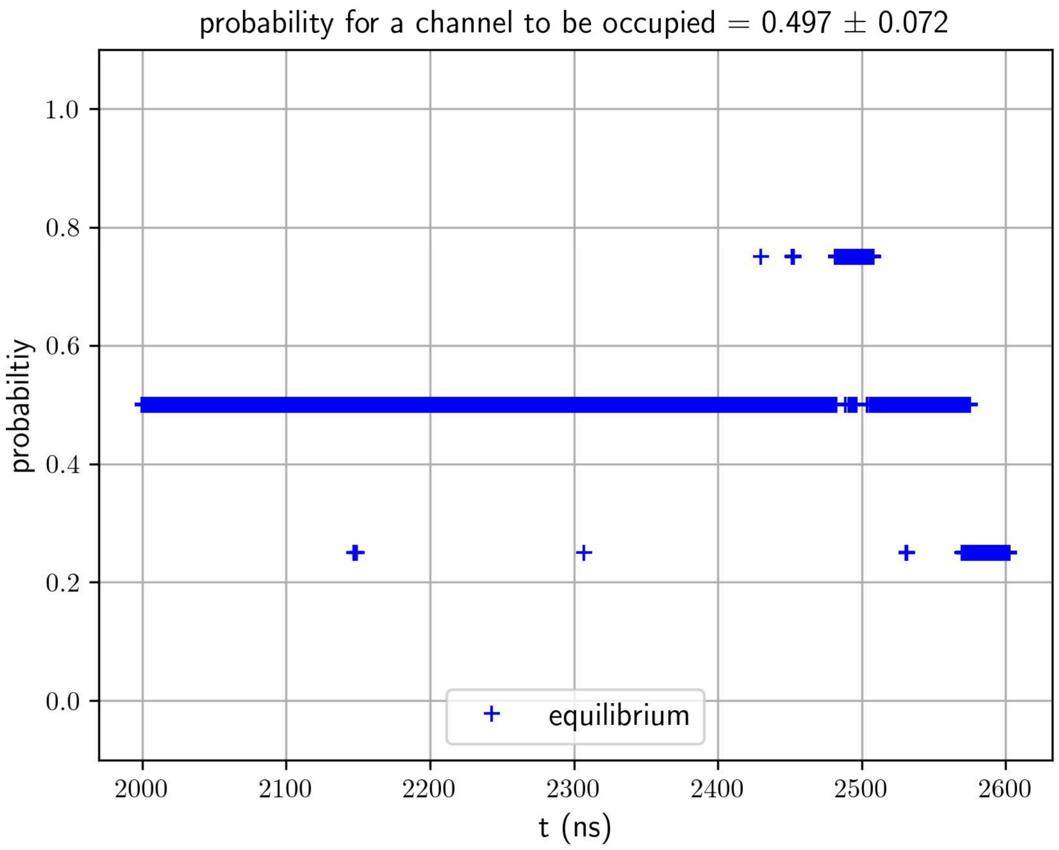

Fig. S5. Glycerol occupancy inside AQP3.

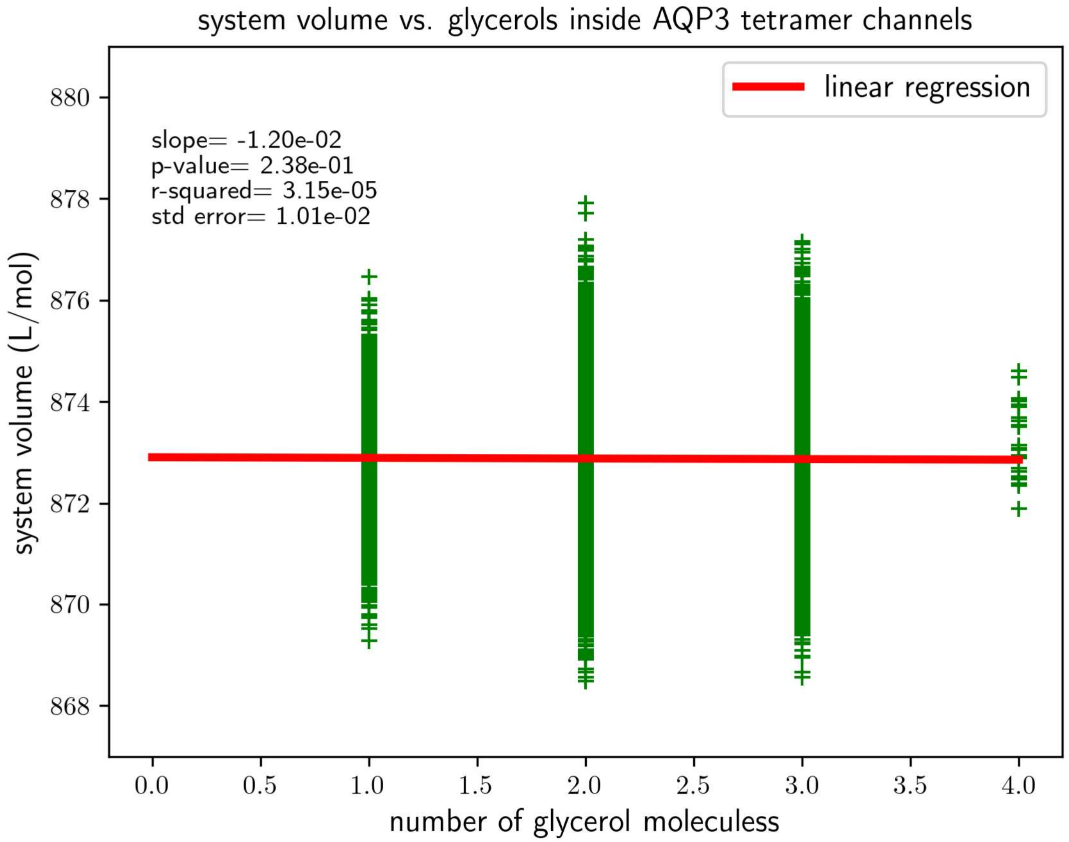

Fig. S6. Volume of System AQP3 vs. glycerol occupancy inside AQP3.

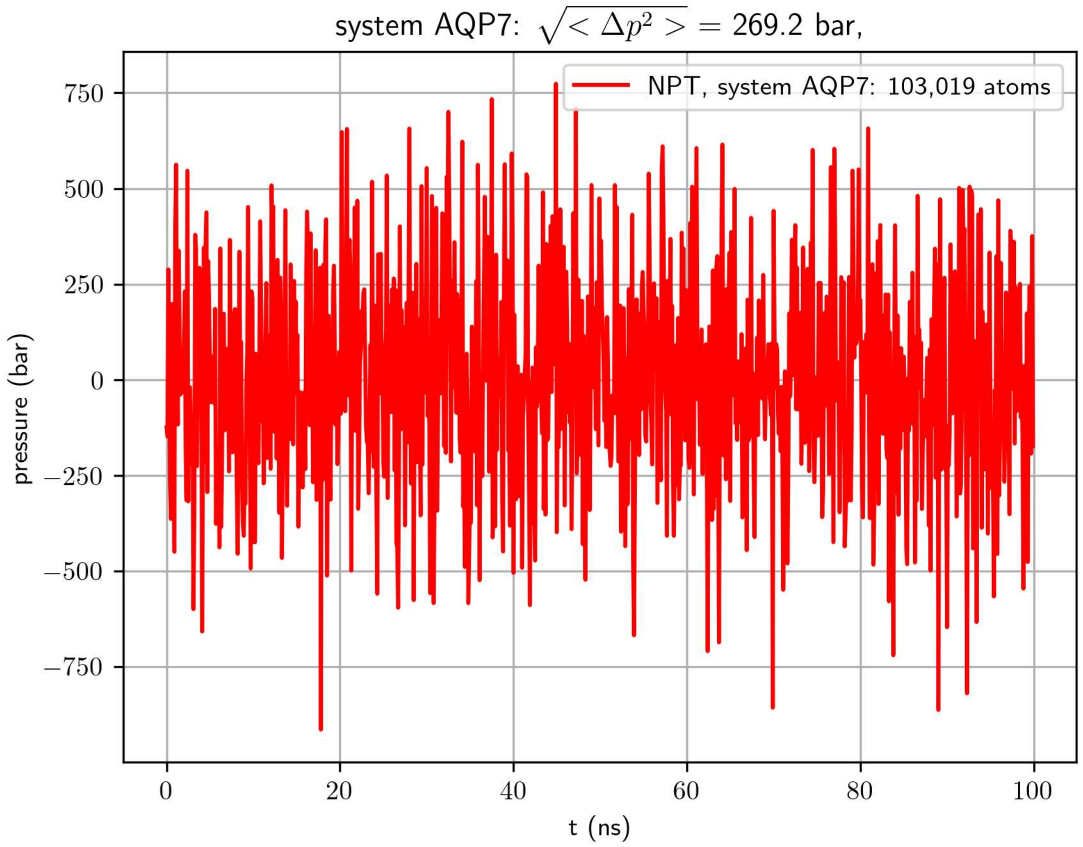

Fig. S7. Pressure fluctuation of System AQP7.

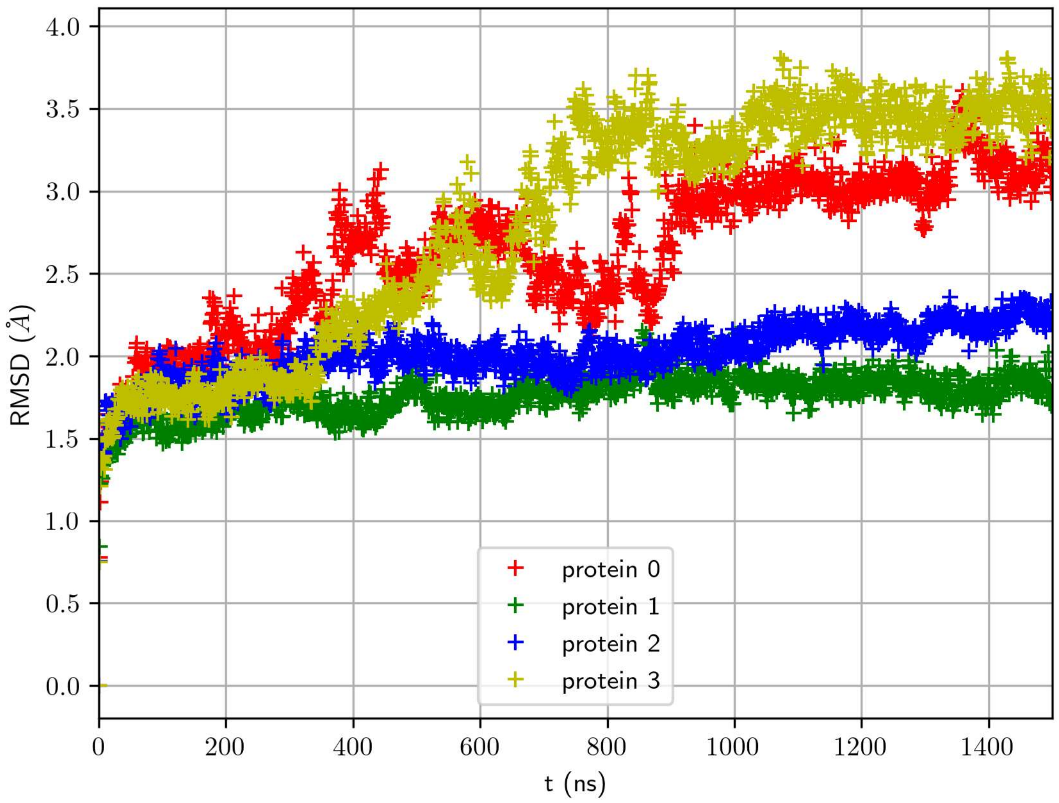

Fig. S8. RMSD of each of the tetramer proteins in System AQP7.

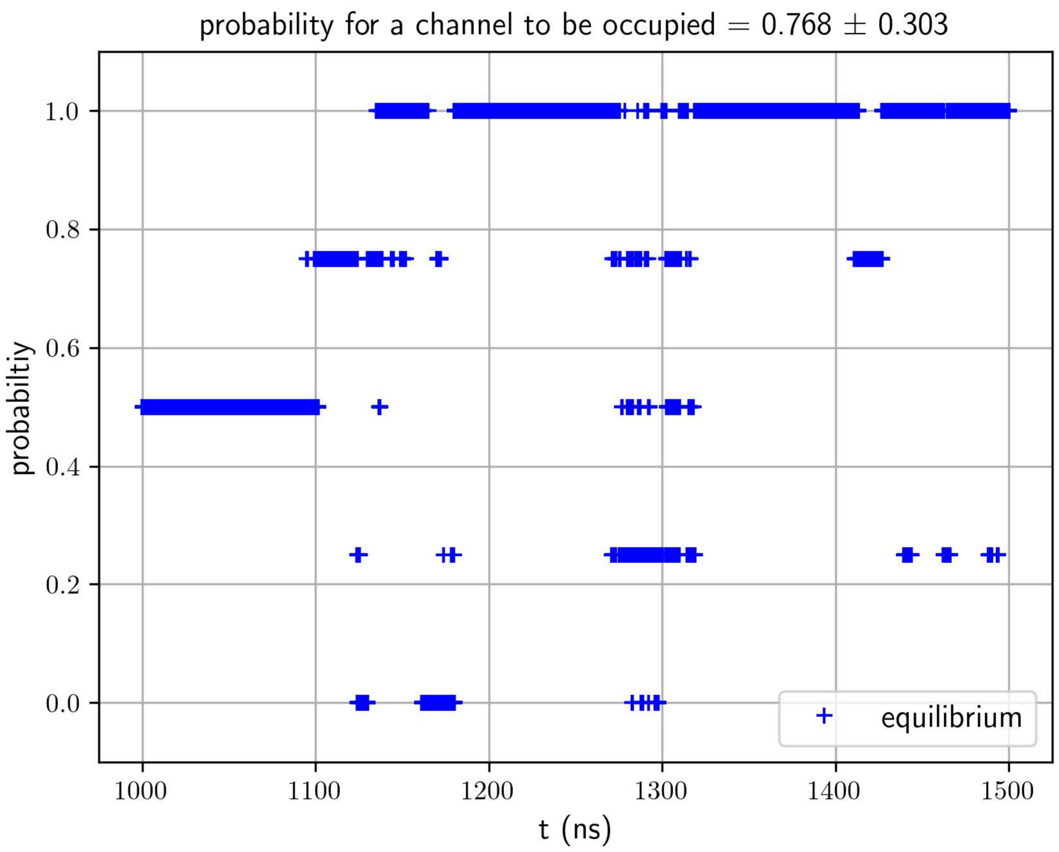

Fig. S9. Glycerol occupancy inside AQP7.

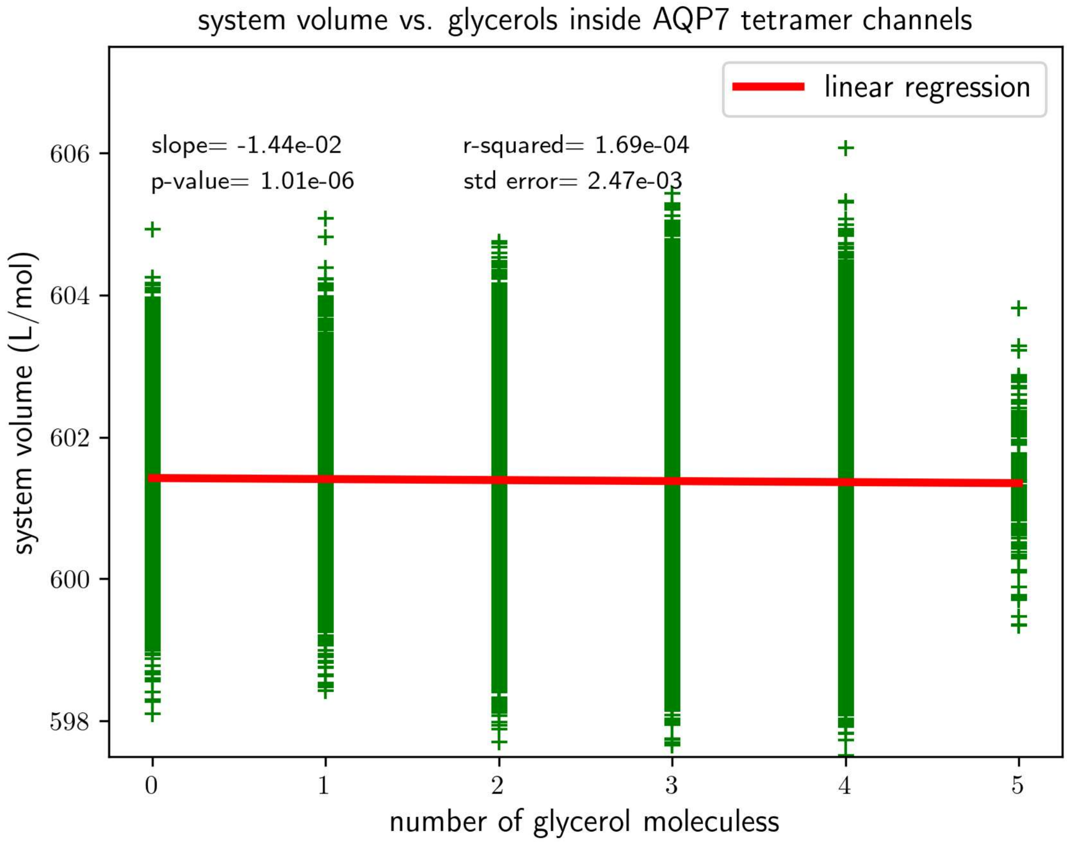

Fig. S10. Volume of System AQP7 vs. glycerol occupancy inside AQP7.

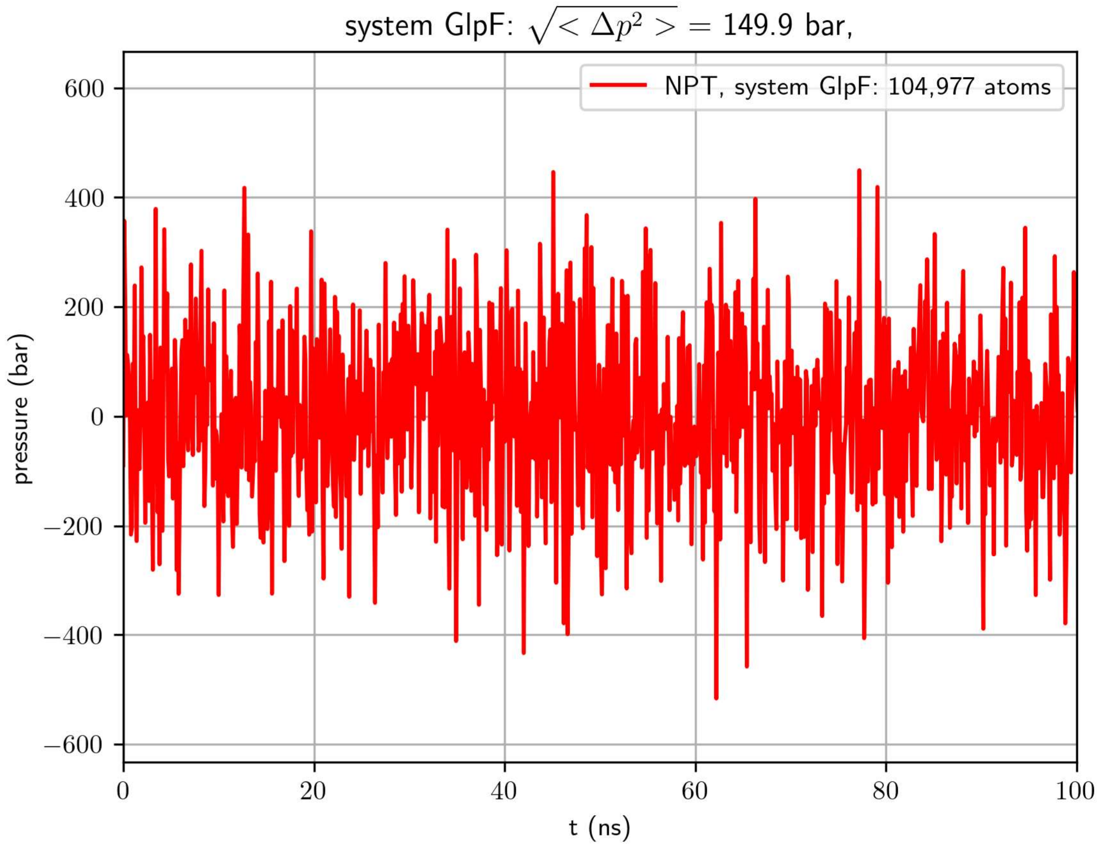

Fig. S11. Pressure fluctuation of System GlpF.

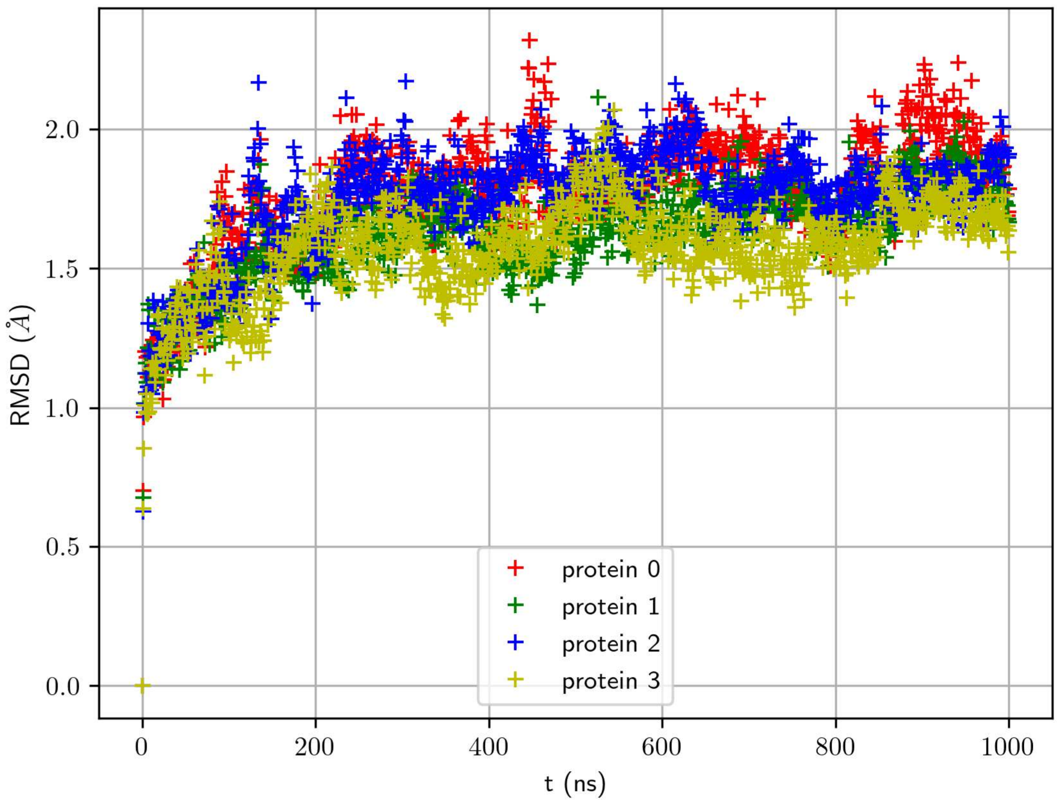

Fig. S12. RMSD of each of the tetramer proteins in System GlpF.

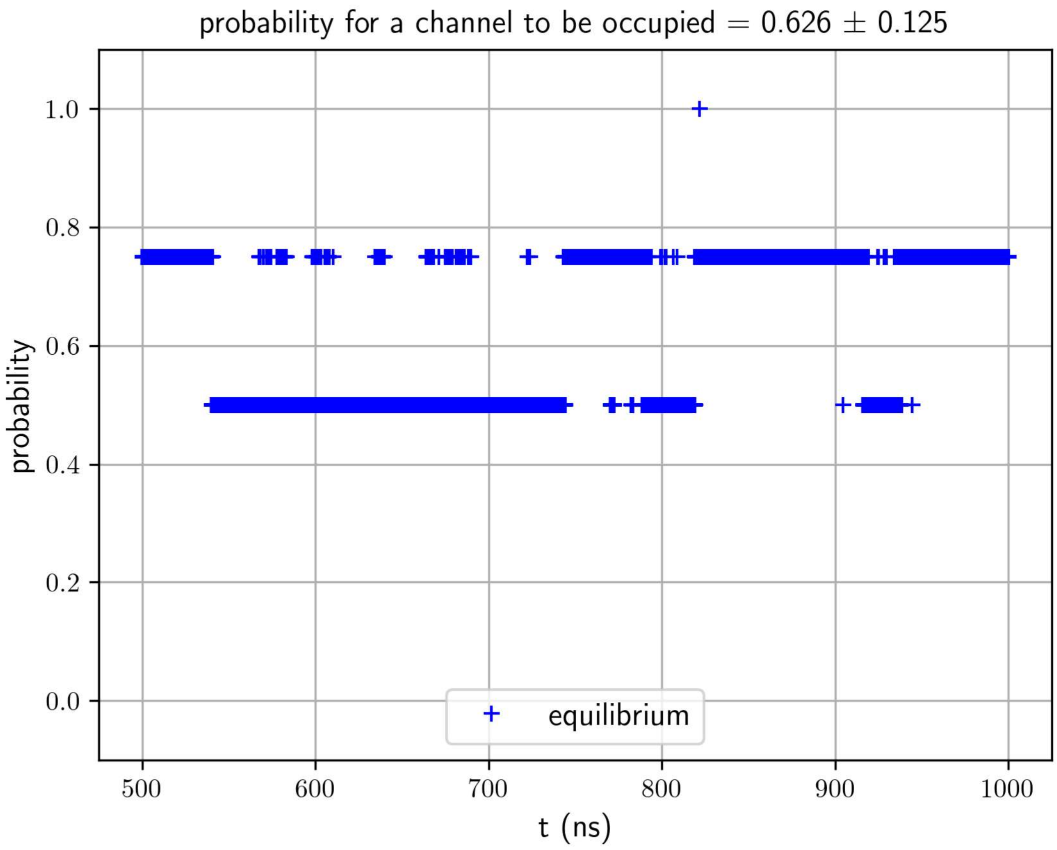

Fig. S13. Glycerol occupancy inside GlpF.

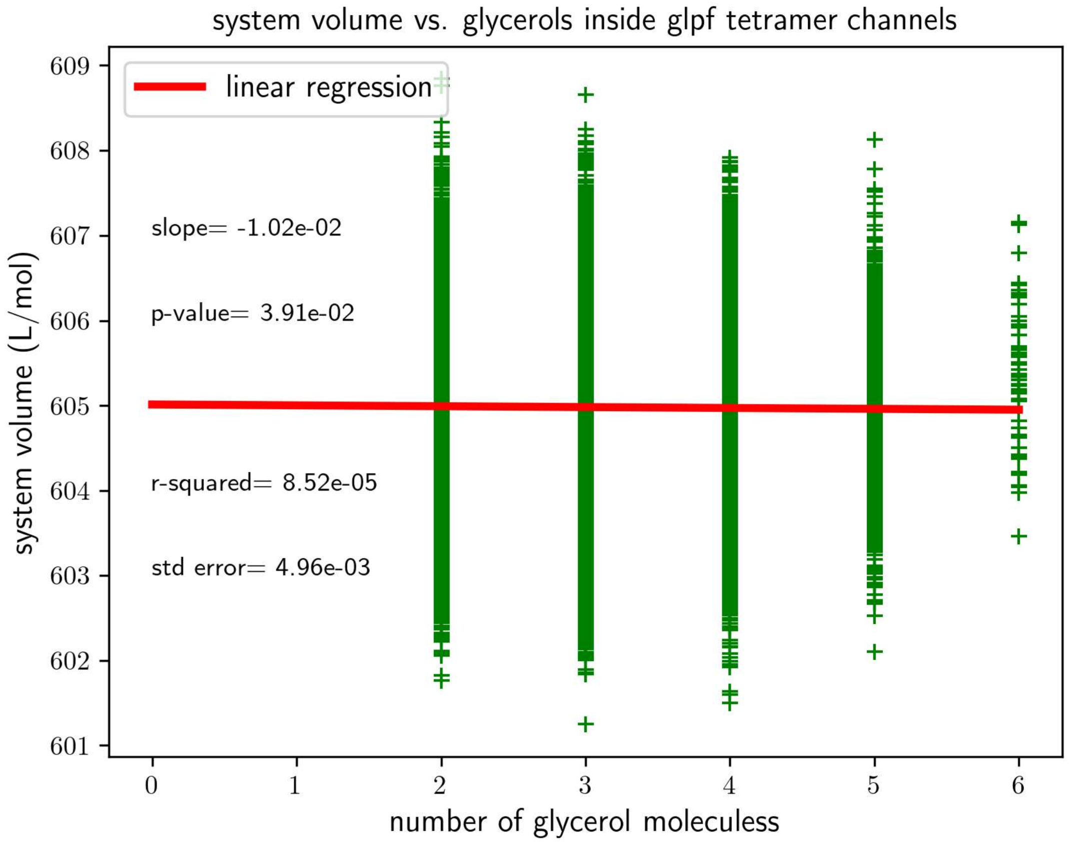

Fig. S14. Volume of System GlpF vs. glycerol occupancy inside GlpF.
